# Crystal structure of bis(μ_2_-tri­phenyl­acetato-κ*O*:κ*O*′)bis(diisobutylaluminium)

**DOI:** 10.1107/S2056989019003396

**Published:** 2019-03-15

**Authors:** Alexander A. Vinogradov, Mikhail E. Minyaev, Konstantin A. Lyssenko, Ilya E. Nifant’ev

**Affiliations:** a A.V. Topchiev Institute of Petrochemical Synthesis, Russian Academy of Sciences, 29 Leninsky prospect, 119991 Moscow, Russian Federation; b G.V. Plekhanov Russian University of Economics, 36 Stremyanny Per., Moscow 117997, Russian Federation; c Chemistry Department, M.V. Lomonosov Moscow State University, 1 Leninskie Gory Str., Building 3, Moscow 119991, Russian Federation

**Keywords:** aluminium, TIBA, tri­phenyl­acetate, coordination compound, crystal structure

## Abstract

Single crystals of the title compound, [Al(iBu)_2_(O_2_CCPh_3_)]_2_ or [Al_2_(C_4_H_9_)_4_(C_20_H_15_O_2_)_2_], have been formed in the reaction between tris­(tetra­hydro­furan)­tris­(tri­phenyl­acetato)­neodymium, [Nd(Ph_3_CCOO)_3_(THF)_3_], and triiso­butyl­aluminium, Al(iBu)_3_, in hexane followed by low-temperature crystallization (243 K) from the reaction mixture. The structure has triclinic (*P* ) symmetry at 120 K. The dimeric complex [Al(iBu)_2_(O_2_CCPh_3_-μ-κ*O*:κ*O*′)]_2_ is located about an inversion centre. The tri­phenyl­acetate ligand displays a μ-κ*O*:κ*O*′-bridging coordination mode, leading to the formation of an octa­gonal Al_2_O_4_C_2_ core. The complex displays H_Ph_⋯C_Ph_ inter­molecular inter­actions.

## Abstract 2

Single crystals of tetra­isobutybis(μ_2_-tri­phenyl­acetato-κ*O*:κ*O*′)dialuminium have been formed in the reaction between tris­(tetra­hydro­furan)­tris­(tri­phenyl­acetato)­neodymium and triiso­butyl­aluminium, Al(iBu)_3_, in hexane followed by low-temperature crystallization (243 K) from the reaction mixture.

## Chemical context   

**Figure 1:**
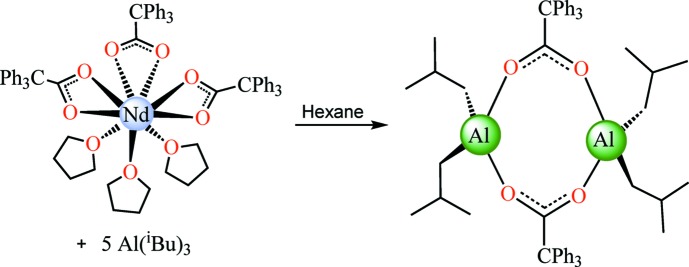
Synthesis of [Al(iBu)_2_(μ-O_2_CCPh_3_)]_2_.

Coordination compounds of lanthanides have attracted considerable attention due to their unique properties as co-catalysts in the stereospecific polymerization of conjugated 1,3-dienes (Anwander, 2002 ▸; Friebe *et al.*, 2006 ▸; Fischbach & Anwander, 2006 ▸; Fischbach *et al.*, 2006 ▸; Kobayashi & Anwander, 2001 ▸; Minyaev *et al.*, 2018*a* ▸,*b* ▸,*c* ▸; Nifant’ev *et al.*, 2013 ▸, 2014 ▸; Zhang *et al.*, 2010 ▸; Kwag, 2002 ▸; Evans *et al.*, 2001 ▸; Evans & Giarikos, 2004 ▸; Roitershtein *et al.*, 2013 ▸, 2019 ▸). The elastomers formed in this process are of fundamental importance with respect to the production of wear-resistant rubbers. Inter­action between organoaluminium and lanthanide complexes usually leads to the formation of Ln–aluminate complexes (*e.g.* see Fischbach *et al.*, 2006 ▸; Roitershtein *et al.*, 2013 ▸, 2019 ▸; Vinogradov *et al.*, 2018 ▸, and references therein), which may be considered as the models for catalytically active species. Sometimes, the second product – an unusual dimeric aluminate complex – forms in this reaction, for instance, when a starting Ln complex contains a bulky tri­phenyl­acetate anion (Roitershtein *et al.*, 2013 ▸) or S/Se-phenyl carbono­thio/seleno­ate ligands (Evans *et al.*, 2006 ▸). This article describes such a product, which was isolated from a reaction between tris­(tetra­hydro­furan)­tris­(tri­phenyl­acetato)­neodymium, [Nd(Ph_3_CCOO)_3_(THF)_3_], and triiso­butyl­aluminium, Al(iBu)_3_ or TIBA, in hexane in a 1:5 ratio, followed by low-temperature crystallization (Fig. 1 ▸).

## Structural commentary   

**Figure 2:**
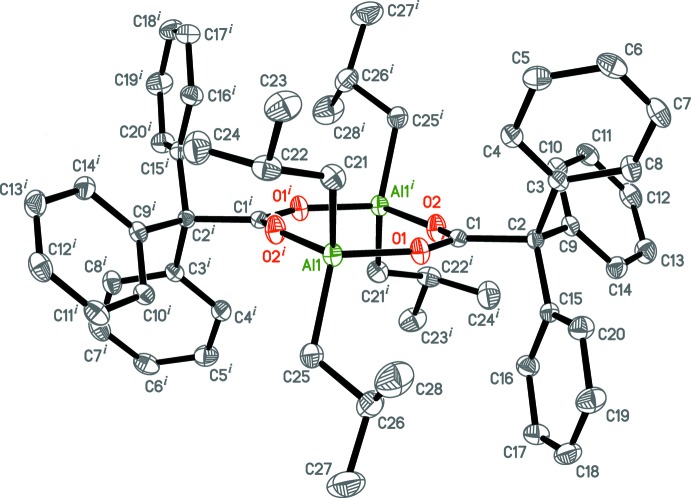
The mol­ecular structure of [Al(iBu)_2_(O_2_CCPh_3_-μ-κ*O*:κ*O*′)]_2_. Displacement ellipsoids are drawn at the 50% probability level and H atoms have been omitted for clarity. [Symmetry code: (i) −*x*, −*y* + 1, −*z* + 1.]

The title compound crystallizes in the triclinic space group *P* 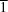. Its asymmetric unit comprises half the dimeric mol­ecule [Al(iBu)_2_(μ-O_2_CCPh_3_)]_2_ (Fig. 2 ▸) located about an inversion centre [symmetry code: (i) −*x* + 1, −*y* + 1, −*z* + 1]. The Al atom adopts a distorted tetra­hedral environment: the O—Al—C and O—Al—O bond angles range from 103.48 (4) (O1—Al—C21) to 108.55 (5)° (O2^i^—Al—C21), whereas the C21—Al—C25 angle is 125.97 (5)°. The tri­phenyl­acetate ligand exhibits a μ_2_-κ*O*:κ*O*′-bridging coordination mode. The C_Ph_—C_Ph_ [1.3788 (17) Å for C5—C6 to 1.4016 (14) Å for C15—C16], C_iBu_—C_iBu_ [1.523 (2) Å for C26—C28 to 1.5381 (16) Å for C21—C22], C1—C2 [1.5473 (13) Å] and C1—C_ipso_ [1.5425 (14) Å for C2—C9 to 1.5455 (14) Å for C2—C3] bond lengths inside the ligands are within the expected ranges. The complex has a nearly flat eight-membered Al_2_O_4_C_2_ core, with the greatest deviations from the plane being 0.0548 (6) Å for the O2 and O2^i^ atoms. The bond angles inside the core are 106.84 (4) (O1—Al—O2^i^), 151.00 (7) (Al—O1—C1), 123.64 (9) (O1—C1—O2) and 156.79 (8)° (C1—O2—Al^i^), summing to a value of 1076.54° for the entire core, which deviates from a flat octa­gon by 3.46°. The Al—*X* bond lengths are given in Table 1 ▸. 20 known crystal structures of [Al*R*_2_(μ-O_2_C*R*′)]_2_ compounds (see §4 below) having the Al_2_O_4_C_2_ core (23 independent core fragments) have Al—*X* bond lengths varying from *ca* 1.77 to 1.86 Å (average 1.82 Å) for Al—O, 1.92–2.00 Å (average 1.96 Å) for Al—C and 1.23–1.29 Å (average 1.26 Å) for C—O bonds. The bond lengths in the title complex (Table 1 ▸) are close to the average values. It might be noted that the Al—O distances in the studied complex are slightly longer than those in alkoxide/aryl­oxide derivatives (the average value for the Al—O distances is 1.76 Å; 946 complexes, 4423 fragments with terminal or μ_2_-bridging *R*O^−^ ligands), but shorter than the Al—O distances in complexes with either Al–O=C*R*_2_ (1.89 Å; 57 complexes; 103 fragments) or Al—O_ether_ fragments (1.98 Å; 471 complexes, 731 fragments) due to different types of Al—O inter­actions, changing from the ion–ion type in the case of Al—O_alk­yl/ar­yl_ bonds to the ion–dipole one in the case of Al—O=C*R*_2_ or Al—O_ether_ fragments.
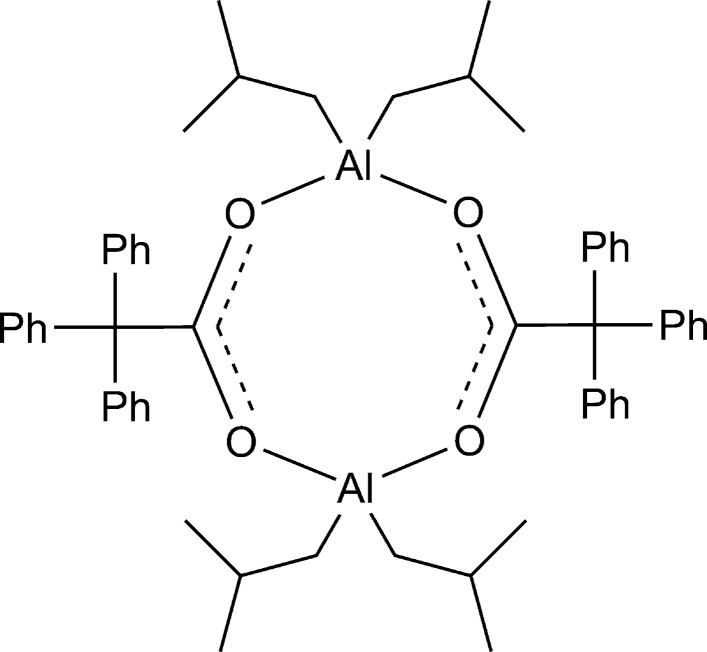


## Supra­molecular features   

**Figure 3:**
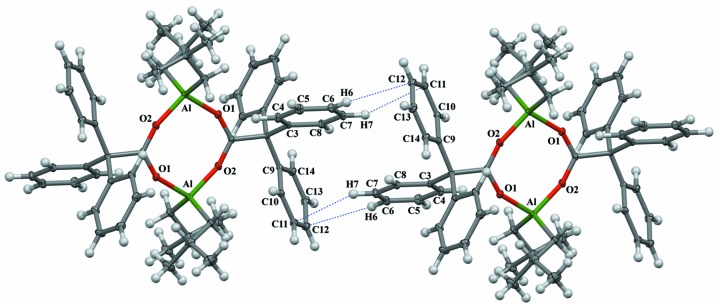
The inter­molecular H_Ph_⋯C_Ph_ inter­actions between neighbouring mol­ecules of [Al(iBu)_2_(O_2_CCPh_3_)]_2_. Displacement ellipsoids for non-H atoms are drawn at the 30% probability level.

The crystal lattice exhibits weak inter­molecular van der Waals contacts between methyl or methyl­ene and aromatic H atoms, with the distances being 2.49 Å for H23*A*⋯H20 and 2.30 Å for H25*A*⋯H12. Two inter­molecular inter­actions involving aromatic H atoms with the π-system of a arene group have been found, *i.e.* 2.89 Å for H6⋯C12 and 2.98 Å for H7⋯C11 (see Table S1 for details). Inter­acting arene rings are located nearly perpendicular to one another, with the corresponding angle between the C3–C8 and C9–C14 planes being 82.83 (3)° (Fig. 3 ▸). The last inter­action type is most likely responsible for the orthogonal orientation for two-thirds of the arene groups in the crystal lattice (see Figs. S1–S3 in the supporting information).

## Database survey   

According to the Cambridge Structural Database (CSD, Version 5.40 with updates, Groom *et al.*, 2016 ▸), there are 20 known crystal structures possessing the Al_2_O_4_C_2_ core and having the [Al*R*_2_(μ-O_2_C*R*′)]_2_ motif, where *R* is alkyl or C_6_F_5_. Records for crystal structures with other *R* groups connected to Al *via* the C atom have not been found in the CSD. 14 complexes have bridging carboxyl­ate ligands, the others have a heteroatom in the α-position (carbamate, seleno­carboxyl­ate and thio­carboxyl­ate ligands).

Complexes of the [Al*RR*’(μ-O_2_C_ar­yl_)]_2_ type are represented by structures with *R* = *R*′ = Me and aryl = Ph (CSD refcode DANMUD; Justyniak *et al.*, 2017 ▸), aryl = 2,4,6-Ph_3_C_6_H_2_ (IZUROK; Dickie *et al.*, 2004 ▸), aryl = 2,4,6-iPr_3_C_6_H_2_ (JEFXEY; Fischbach *et al.*, 2006 ▸); *R* = *R*′ = *tert*-butyl and aryl = Ph (RITQUG; Bethley *et al.*, 1997 ▸), aryl = 2-NMe_2_C_6_H_4_ (MIJZEK; Branch *et al.*, 2001 ▸), and *R* = Me, *R*′ = C(SiMe_3_)_3_ and aryl = 4-MeC_6_H_4_ (OXUZUD; Kalita *et al.*, 2011 ▸). Two complexes with several Al_2_O_4_C_2_ skeleton fragments, containing 2,2′-O_2_C–C_6_H_4_–C_6_H_4_–CO_2_ di­carboxyl­ate ligands have *R* = Et (RUJCIJ; two fragments) and *R* = isobutyl (iBu) (RUJCOP; three fragments) (Ziemkowska *et al.*, 2009 ▸). Three [Al*R*_2_(μ-O_2_CC*X*_3_)]_2_ complexes with a substituted acetate anion possess *R* = Et and C*X*_3_ = CPh_3_ (RIJVEN; Roitershtein *et al.*, 2013 ▸; this complex has a very similar structure compared with that described herein but a ‘less flat’ core), and *R* = *tert*-butyl, and C*X*_3_ = CH_2_Ph, *tert*-butyl and CH_2_OC_2_H_4_OCH_3_ (RITRAN, RITQOA and RITRER; Bethley *et al.*, 1997 ▸). The other complexes are [Al(iBu)_2_(μ_2_-O_2_C*X*)]_2_, with *X* = –C_4_H(CH_3_)_2_Zr(η^5^-C_5_Me_5_)_2_ (OBOLIB; Burlakov *et al.*, 2004 ▸), [Al(C_6_F_5_)_2_(μ-O_2_CC_6_F_5_)]_2_ (ZIGGON; Ménard *et al.*, 2013 ▸), [AlMe_2_(μ-O_2_C*E*Ph)]_2_ (*E* = S for YEBKAS and *E* = Se for YEBKIA; Evans *et al.*, 2006 ▸), [Al*R*_2_(μ-O_2_CN*X*_2_)]_2_, with *R* = iBu (NACYUN; Kennedy *et al.*, 2010 ▸), *tert*-Bu (OFELIW; Hengesbach *et al.*, 2013 ▸) and Me [XAPKEH (Zijlstra *et al.*, 2017 ▸) and ZIQLEQ (Chang *et al.*, 1995 ▸)].

Based on an analysis of the listed structures, the Al_2_O_4_C_2_ core is quite flexible and its conformation (from flat to chair-like) depends greatly on various inter­actions within the complex, including nonvalence ones. See also related *ab initio* calculations in the literature (Bethley *et al.*, 1997 ▸).

## Synthesis and crystallization   

All synthetic manipulations were performed under a purified argon atmosphere, using Schlenk glassware, dry-box techniques and absolute solvents. The C/H elemental analysis was performed with a PerkinElmer 2400 Series II elemental analyzer. Hexane was distilled from Na/K alloy. The complex [Nd(Ph_3_CCOO)_3_(THF)_3_] was prepared according to a previously published method (Roitershtein *et al.*, 2013 ▸).

A solution of Al(iBu)_3_ in hexane (1 *M*, 0.5 ml, 0.5 mmol) was added dropwise to a suspension of [Nd(Ph_3_CCOO)_3_(THF)_3_] (0.122 g, 0.1 mmol) in 15 ml of hexane at room temperature. The suspension dissolved within a few minutes upon addition. The resulting solution was stirred overnight at room temperature. Crystals of [Al(iBu)_2_(Ph_3_CCOO)]_2_ were isolated from the reaction mixture by crystallization at 243 K for 2 d. The mother liquor was deca­nted and crystals were dried under dynamic vacuum. The yield was 56 mg (0.065 mmol, 43% based on the Ph_3_CCO_2_^−^ ligand or 26% based on Al). Calculated for C_56_H_66_Al_2_O_4_ (%): C 78.48, H 7.76; found: C 78.17, H 8.01.

## Refinement   

Crystal data, data collection and structure refinement details are summarized in Table 2 ▸. The H atoms were positioned geometrically (C—H = 0.95 Å for aromatic, 0.98 Å for methyl, 0.99 Å for methyl­ene and 1.00 Å for methine H atoms) and refined as riding atoms with relative isotropic displacement parameters *U*_iso_(H) = 1.5*U*_eq_(C) for methyl H atoms and 1.2*U*_eq_(C) otherwise. A rotating group model was applied for methyl groups.

## Supplementary Material

Crystal structure: contains datablock(s) global. DOI: 10.1107/S2056989019003396/tx2010sup1.cif

C...H interactions and packing plots. DOI: 10.1107/S2056989019003396/tx2010sup4.pdf

CCDC reference: 1902149

Additional supporting information:  crystallographic information; 3D view; checkCIF report

## Figures and Tables

**Table 1:** Selected bond lengths (Å)

Al—O1	1.8269 (8)	Al—C25	1.9611 (12)
Al—O2^i^	1.8212 (8)	O1—C1	1.2579 (12)
Al—C21	1.9639 (12)	O2—C1	1.2518 (12)

Symmetry code: (i) 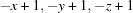.

**Table 2:** Experimental details

Crystal data	
Chemical formula	[Al_2_(C_4_H_9_)_4_(C_20_H_15_O_2_)_2_]
*M*_r_	857.04
Crystal system, space group	Triclinic, *P* 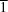
Temperature (K)	120
*a*, *b*, *c* (Å)	9.2839 (3), 12.0281 (4), 12.5999 (4)
α, β, γ (°)	108.790 (1), 109.143 (1), 91.866 (1)
*V* (Å^3^)	1243.25 (7)
*Z*	1
Radiation type	Mo *K*α
μ (mm^−1^)	0.10
Crystal size (mm)	0.32 × 0.21 × 0.18

Data collection	
Diffractometer	Bruker SMART APEXII
Absorption correction	Multi-scan (*SADABS*; Krause *et al.*, 2015 ▸)
*T*_min_, *T*_max_	0.684, 0.747
No. of measured, independent and observed [*I* > 2σ(*I*)] reflections	16191, 7242, 6063
*R*_int_	0.016
(sin θ/λ)_max_ (Å^−1^)	0.703

Refinement	
*R*[*F* ^2^ > 2σ(*F* ^2^)], *wR*(*F* ^2^), *S*	0.038, 0.105, 1.02
No. of reflections	7242
No. of parameters	284
H-atom treatment	H-atom parameters constrained
Δρ_max_, Δρ_min_ (e Å^−3^)	0.41, −0.23

Computer programs: *APEX2* (Bruker, 2008 ▸), *SHELXS97* (Sheldrick, 2008 ▸), *SHELXL2017* (Sheldrick, 2015 ▸), *SHELXTL* (Sheldrick, 2008 ▸), *Mercury* (Macrae *et al.*, 2008 ▸) and *publCIF* (Westrip, 2010 ▸).

## supplementary crystallographic information

### Crystal data

**Table:**

[Al_2_(C_4_H_9_)_4_(C_20_H_15_O_2_)_2_]	*Z* = 1
*M**_r_* = 857.04	*F*(000) = 460
Triclinic, *P*1	*D*_x_ = 1.145 Mg m^−^^3^
*a* = 9.2839 (3) Å	Mo *K*α radiation, λ = 0.71073 Å
*b* = 12.0281 (4) Å	Cell parameters from 4293 reflections
*c* = 12.5999 (4) Å	θ = 2.2–28.3°
α = 108.790 (1)°	µ = 0.10 mm^−^^1^
β = 109.143 (1)°	*T* = 120 K
γ = 91.866 (1)°	Block, colourless
*V* = 1243.25 (7) Å^3^	0.32 × 0.21 × 0.18 mm

### Data collection

**Table:**

Bruker SMART APEXII diffractometer	7242 independent reflections
Radiation source: fine-focus sealed tube	6063 reflections with *I* > 2σ(*I*)
Graphite monochromator	*R*_int_ = 0.016
ω scans	θ_max_ = 30.0°, θ_min_ = 1.8°
Absorption correction: multi-scan (SADABS; Krause *et al.*, 2015)	*h* = −13→12
*T*_min_ = 0.684, *T*_max_ = 0.747	*k* = −16→16
16191 measured reflections	*l* = −17→17

### Refinement

**Table:**

Refinement on *F*^2^	Primary atom site location: structure-invariant direct methods
Least-squares matrix: full	Secondary atom site location: difference Fourier map
*R*[*F*^2^ > 2σ(*F*^2^)] = 0.038	Hydrogen site location: inferred from neighbouring sites
*wR*(*F*^2^) = 0.105	H-atom parameters constrained
*S* = 1.01	*w* = 1/[σ^2^(*F*_o_^2^) + (0.0485*P*)^2^ + 0.4816*P*] where *P* = (*F*_o_^2^ + 2*F*_c_^2^)/3
7242 reflections	(Δ/σ)_max_ = 0.001
284 parameters	Δρ_max_ = 0.41 e Å^−^^3^
0 restraints	Δρ_min_ = −0.23 e Å^−^^3^

### Special details

**Table:**

Experimental. moisture and air sensitive
Geometry. All esds (except the esd in the dihedral angle between two l.s. planes) are estimated using the full covariance matrix. The cell esds are taken into account individually in the estimation of esds in distances, angles and torsion angles; correlations between esds in cell parameters are only used when they are defined by crystal symmetry. An approximate (isotropic) treatment of cell esds is used for estimating esds involving l.s. planes.
Refinement. Refinement of F^2^ against ALL reflections. The weighted R-factor wR and goodness of fit S are based on F^2^, conventional R-factors R are based on F, with F set to zero for negative F^2^. The threshold expression of F^2^ > 2sigma(F^2^) is used only for calculating R-factors(gt) etc. and is not relevant to the choice of reflections for refinement. R-factors based on F^2^ are statistically about twice as large as those based on F, and R- factors based on ALL data will be even larger.

### Fractional atomic coordinates and isotropic or equivalent isotropic displacement parameters (Å^2^)

**Table:**

	*x*	*y*	*z*	*U*_iso_*/*U*_eq_	
Al	0.36034 (4)	0.60789 (3)	0.40800 (3)	0.01642 (8)	
O1	0.46295 (9)	0.65487 (7)	0.56994 (7)	0.02070 (16)	
O2	0.59680 (10)	0.53977 (7)	0.65814 (7)	0.02258 (17)	
C1	0.55346 (11)	0.63769 (9)	0.66037 (9)	0.01473 (18)	
C2	0.60602 (11)	0.74392 (8)	0.78064 (8)	0.01395 (17)	
C3	0.70126 (11)	0.84711 (9)	0.77322 (9)	0.01552 (18)	
C4	0.72549 (13)	0.84652 (10)	0.66985 (10)	0.0214 (2)	
H4	0.679269	0.780431	0.597423	0.026*	
C5	0.81694 (14)	0.94188 (11)	0.67113 (11)	0.0264 (2)	
H5	0.831675	0.940322	0.599551	0.032*	
C6	0.88595 (13)	1.03822 (10)	0.77555 (11)	0.0255 (2)	
H6	0.948184	1.102928	0.776255	0.031*	
C7	0.86365 (14)	1.03974 (10)	0.87945 (11)	0.0254 (2)	
H7	0.910835	1.105845	0.951716	0.030*	
C8	0.77282 (13)	0.94532 (9)	0.87861 (10)	0.0213 (2)	
H8	0.758995	0.947335	0.950612	0.026*	
C9	0.71189 (11)	0.70895 (9)	0.88401 (9)	0.01570 (18)	
C10	0.84898 (12)	0.66876 (9)	0.87587 (9)	0.01762 (19)	
H10	0.872291	0.660624	0.805990	0.021*	
C11	0.95149 (13)	0.64058 (10)	0.96847 (10)	0.0219 (2)	
H11	1.043453	0.612413	0.961155	0.026*	
C12	0.91965 (14)	0.65356 (10)	1.07182 (10)	0.0245 (2)	
H12	0.989225	0.633819	1.135044	0.029*	
C13	0.78621 (14)	0.69535 (11)	1.08199 (10)	0.0249 (2)	
H13	0.764773	0.705090	1.152906	0.030*	
C14	0.68228 (13)	0.72347 (10)	0.98882 (10)	0.0208 (2)	
H14	0.591151	0.752588	0.997035	0.025*	
C15	0.45248 (11)	0.77495 (9)	0.79625 (9)	0.01574 (18)	
C16	0.35242 (12)	0.68609 (10)	0.79768 (10)	0.0204 (2)	
H16	0.382480	0.610353	0.792614	0.025*	
C17	0.20989 (13)	0.70739 (11)	0.80642 (11)	0.0243 (2)	
H17	0.143354	0.646379	0.807484	0.029*	
C18	0.16423 (13)	0.81765 (11)	0.81363 (11)	0.0260 (2)	
H18	0.066942	0.832509	0.819938	0.031*	
C19	0.26197 (14)	0.90553 (11)	0.81151 (12)	0.0276 (2)	
H19	0.231175	0.980971	0.816053	0.033*	
C20	0.40547 (13)	0.88454 (10)	0.80277 (10)	0.0219 (2)	
H20	0.471338	0.945698	0.801290	0.026*	
C21	0.46133 (13)	0.72019 (10)	0.36084 (10)	0.0222 (2)	
H21A	0.469150	0.800830	0.418442	0.027*	
H21C	0.568096	0.703514	0.372745	0.027*	
C22	0.39213 (13)	0.72538 (10)	0.23396 (11)	0.0238 (2)	
H22	0.281201	0.734727	0.218042	0.029*	
C23	0.47318 (17)	0.83216 (12)	0.22378 (13)	0.0320 (3)	
H23A	0.460235	0.905703	0.280219	0.048*	
H23B	0.427888	0.832215	0.141750	0.048*	
H23C	0.583350	0.826987	0.242832	0.048*	
C24	0.39865 (17)	0.61094 (12)	0.13896 (12)	0.0333 (3)	
H24A	0.357500	0.618086	0.059590	0.050*	
H24B	0.336713	0.544191	0.140295	0.050*	
H24C	0.505921	0.596939	0.155904	0.050*	
C25	0.13991 (13)	0.58192 (10)	0.38202 (10)	0.0220 (2)	
H25A	0.081171	0.575283	0.298399	0.026*	
H25B	0.116166	0.504248	0.388900	0.026*	
C26	0.07847 (14)	0.67528 (12)	0.46520 (11)	0.0279 (2)	
H26	0.134767	0.679617	0.549380	0.033*	
C27	−0.09381 (17)	0.63988 (16)	0.43580 (15)	0.0430 (3)	
H27A	−0.128031	0.698006	0.493736	0.064*	
H27B	−0.111633	0.561008	0.440224	0.064*	
H27C	−0.152073	0.637961	0.354626	0.064*	
C28	0.1090 (2)	0.79795 (14)	0.45938 (16)	0.0440 (4)	
H28A	0.068374	0.855379	0.513088	0.066*	
H28B	0.057855	0.795392	0.376941	0.066*	
H28C	0.220363	0.822015	0.484379	0.066*	

### Atomic displacement parameters (Å^2^)

**Table:**

	*U*^11^	*U*^22^	*U*^33^	*U*^12^	*U*^13^	*U*^23^
Al	0.01816 (15)	0.01465 (14)	0.01484 (14)	0.00357 (11)	0.00382 (11)	0.00514 (11)
O1	0.0225 (4)	0.0188 (4)	0.0153 (3)	0.0026 (3)	0.0020 (3)	0.0038 (3)
O2	0.0279 (4)	0.0149 (3)	0.0192 (4)	0.0047 (3)	0.0044 (3)	0.0026 (3)
C1	0.0135 (4)	0.0144 (4)	0.0153 (4)	−0.0002 (3)	0.0057 (3)	0.0037 (3)
C2	0.0141 (4)	0.0127 (4)	0.0134 (4)	0.0015 (3)	0.0043 (3)	0.0031 (3)
C3	0.0140 (4)	0.0140 (4)	0.0173 (4)	0.0019 (3)	0.0044 (3)	0.0052 (4)
C4	0.0213 (5)	0.0208 (5)	0.0196 (5)	−0.0016 (4)	0.0074 (4)	0.0041 (4)
C5	0.0273 (6)	0.0273 (6)	0.0286 (6)	−0.0002 (4)	0.0135 (5)	0.0118 (5)
C6	0.0214 (5)	0.0207 (5)	0.0349 (6)	−0.0009 (4)	0.0080 (5)	0.0128 (5)
C7	0.0256 (5)	0.0163 (5)	0.0260 (5)	−0.0035 (4)	0.0023 (4)	0.0047 (4)
C8	0.0248 (5)	0.0175 (5)	0.0177 (5)	−0.0007 (4)	0.0047 (4)	0.0045 (4)
C9	0.0158 (4)	0.0137 (4)	0.0148 (4)	0.0001 (3)	0.0031 (3)	0.0039 (3)
C10	0.0167 (4)	0.0163 (4)	0.0181 (5)	0.0006 (3)	0.0048 (4)	0.0055 (4)
C11	0.0175 (5)	0.0202 (5)	0.0251 (5)	0.0024 (4)	0.0030 (4)	0.0088 (4)
C12	0.0247 (5)	0.0233 (5)	0.0212 (5)	0.0010 (4)	0.0001 (4)	0.0107 (4)
C13	0.0281 (6)	0.0286 (6)	0.0167 (5)	0.0015 (4)	0.0055 (4)	0.0094 (4)
C14	0.0208 (5)	0.0232 (5)	0.0178 (5)	0.0036 (4)	0.0065 (4)	0.0067 (4)
C15	0.0147 (4)	0.0170 (4)	0.0140 (4)	0.0022 (3)	0.0048 (3)	0.0038 (3)
C16	0.0191 (5)	0.0191 (5)	0.0231 (5)	0.0018 (4)	0.0082 (4)	0.0069 (4)
C17	0.0196 (5)	0.0278 (6)	0.0267 (5)	0.0009 (4)	0.0106 (4)	0.0092 (4)
C18	0.0201 (5)	0.0343 (6)	0.0262 (6)	0.0083 (4)	0.0121 (4)	0.0099 (5)
C19	0.0270 (6)	0.0255 (6)	0.0351 (6)	0.0120 (5)	0.0155 (5)	0.0117 (5)
C20	0.0223 (5)	0.0190 (5)	0.0269 (5)	0.0049 (4)	0.0113 (4)	0.0086 (4)
C21	0.0228 (5)	0.0200 (5)	0.0234 (5)	0.0027 (4)	0.0069 (4)	0.0083 (4)
C22	0.0212 (5)	0.0261 (5)	0.0284 (6)	0.0040 (4)	0.0086 (4)	0.0156 (5)
C23	0.0396 (7)	0.0267 (6)	0.0412 (7)	0.0074 (5)	0.0217 (6)	0.0190 (5)
C24	0.0438 (7)	0.0290 (6)	0.0258 (6)	−0.0037 (5)	0.0118 (5)	0.0096 (5)
C25	0.0214 (5)	0.0266 (5)	0.0176 (5)	0.0038 (4)	0.0058 (4)	0.0085 (4)
C26	0.0269 (6)	0.0367 (6)	0.0227 (5)	0.0115 (5)	0.0108 (5)	0.0113 (5)
C27	0.0307 (7)	0.0603 (10)	0.0474 (8)	0.0159 (7)	0.0223 (6)	0.0218 (7)
C28	0.0497 (9)	0.0339 (7)	0.0557 (9)	0.0175 (6)	0.0288 (8)	0.0140 (7)

### Geometric parameters (Å, º)

**Table:**

Al—O1	1.8269 (8)	C16—C17	1.3887 (15)
Al—O2^i^	1.8212 (8)	C16—H16	0.9500
Al—C21	1.9639 (12)	C17—C18	1.3896 (17)
Al—C25	1.9611 (12)	C17—H17	0.9500
O1—C1	1.2579 (12)	C18—C19	1.3831 (18)
O2—C1	1.2518 (12)	C18—H18	0.9500
C1—C2	1.5473 (13)	C19—C20	1.3968 (16)
C2—C9	1.5425 (14)	C19—H19	0.9500
C2—C15	1.5431 (14)	C20—H20	0.9500
C2—C3	1.5455 (14)	C21—C22	1.5381 (16)
C3—C4	1.3905 (14)	C21—H21A	0.9900
C3—C8	1.3999 (14)	C21—H21C	0.9900
C4—C5	1.3973 (15)	C22—C24	1.5241 (18)
C4—H4	0.9500	C22—C23	1.5293 (16)
C5—C6	1.3788 (17)	C22—H22	1.0000
C5—H5	0.9500	C23—H23A	0.9800
C6—C7	1.3855 (17)	C23—H23B	0.9800
C6—H6	0.9500	C23—H23C	0.9800
C7—C8	1.3875 (15)	C24—H24A	0.9800
C7—H7	0.9500	C24—H24B	0.9800
C8—H8	0.9500	C24—H24C	0.9800
C9—C14	1.3938 (14)	C25—C26	1.5363 (16)
C9—C10	1.3992 (14)	C25—H25A	0.9900
C10—C11	1.3890 (14)	C25—H25B	0.9900
C10—H10	0.9500	C26—C28	1.523 (2)
C11—C12	1.3900 (17)	C26—C27	1.5304 (19)
C11—H11	0.9500	C26—H26	1.0000
C12—C13	1.3809 (17)	C27—H27A	0.9800
C12—H12	0.9500	C27—H27B	0.9800
C13—C14	1.3992 (15)	C27—H27C	0.9800
C13—H13	0.9500	C28—H28A	0.9800
C14—H14	0.9500	C28—H28B	0.9800
C15—C20	1.3878 (14)	C28—H28C	0.9800
C15—C16	1.4016 (14)		
			
O2^i^—Al—O1	106.84 (4)	C16—C17—H17	119.9
O2^i^—Al—C25	104.41 (5)	C18—C17—H17	119.9
O1—Al—C25	106.37 (4)	C19—C18—C17	119.28 (10)
O2^i^—Al—C21	108.55 (5)	C19—C18—H18	120.4
O1—Al—C21	103.48 (4)	C17—C18—H18	120.4
C25—Al—C21	125.97 (5)	C18—C19—C20	120.70 (11)
C1—O1—Al	151.00 (7)	C18—C19—H19	119.7
C1—O2—Al^i^	156.79 (8)	C20—C19—H19	119.7
O2—C1—O1	123.64 (9)	C15—C20—C19	120.45 (10)
O2—C1—C2	119.65 (9)	C15—C20—H20	119.8
O1—C1—C2	116.65 (9)	C19—C20—H20	119.8
C9—C2—C15	112.68 (8)	C22—C21—Al	120.48 (8)
C9—C2—C3	106.65 (8)	C22—C21—H21A	107.2
C15—C2—C3	113.13 (8)	Al—C21—H21A	107.2
C9—C2—C1	110.97 (8)	C22—C21—H21C	107.2
C15—C2—C1	103.23 (8)	Al—C21—H21C	107.2
C3—C2—C1	110.25 (8)	H21A—C21—H21C	106.8
C4—C3—C8	117.96 (9)	C24—C22—C23	110.11 (10)
C4—C3—C2	124.20 (9)	C24—C22—C21	111.38 (10)
C8—C3—C2	117.79 (9)	C23—C22—C21	111.41 (10)
C3—C4—C5	120.85 (10)	C24—C22—H22	107.9
C3—C4—H4	119.6	C23—C22—H22	107.9
C5—C4—H4	119.6	C21—C22—H22	107.9
C6—C5—C4	120.45 (11)	C22—C23—H23A	109.5
C6—C5—H5	119.8	C22—C23—H23B	109.5
C4—C5—H5	119.8	H23A—C23—H23B	109.5
C5—C6—C7	119.35 (10)	C22—C23—H23C	109.5
C5—C6—H6	120.3	H23A—C23—H23C	109.5
C7—C6—H6	120.3	H23B—C23—H23C	109.5
C6—C7—C8	120.41 (10)	C22—C24—H24A	109.5
C6—C7—H7	119.8	C22—C24—H24B	109.5
C8—C7—H7	119.8	H24A—C24—H24B	109.5
C7—C8—C3	120.97 (10)	C22—C24—H24C	109.5
C7—C8—H8	119.5	H24A—C24—H24C	109.5
C3—C8—H8	119.5	H24B—C24—H24C	109.5
C14—C9—C10	118.49 (9)	C26—C25—Al	117.87 (8)
C14—C9—C2	122.79 (9)	C26—C25—H25A	107.8
C10—C9—C2	118.56 (9)	Al—C25—H25A	107.8
C11—C10—C9	120.97 (10)	C26—C25—H25B	107.8
C11—C10—H10	119.5	Al—C25—H25B	107.8
C9—C10—H10	119.5	H25A—C25—H25B	107.2
C10—C11—C12	120.06 (10)	C28—C26—C27	110.38 (12)
C10—C11—H11	120.0	C28—C26—C25	111.39 (11)
C12—C11—H11	120.0	C27—C26—C25	111.31 (11)
C13—C12—C11	119.55 (10)	C28—C26—H26	107.9
C13—C12—H12	120.2	C27—C26—H26	107.9
C11—C12—H12	120.2	C25—C26—H26	107.9
C12—C13—C14	120.62 (11)	C26—C27—H27A	109.5
C12—C13—H13	119.7	C26—C27—H27B	109.5
C14—C13—H13	119.7	H27A—C27—H27B	109.5
C9—C14—C13	120.28 (10)	C26—C27—H27C	109.5
C9—C14—H14	119.9	H27A—C27—H27C	109.5
C13—C14—H14	119.9	H27B—C27—H27C	109.5
C20—C15—C16	118.53 (9)	C26—C28—H28A	109.5
C20—C15—C2	123.12 (9)	C26—C28—H28B	109.5
C16—C15—C2	118.27 (9)	H28A—C28—H28B	109.5
C17—C16—C15	120.81 (10)	C26—C28—H28C	109.5
C17—C16—H16	119.6	H28A—C28—H28C	109.5
C15—C16—H16	119.6	H28B—C28—H28C	109.5
C16—C17—C18	120.23 (11)		
			
O2^i^—Al—O1—C1	3.48 (16)	C15—C2—C9—C10	174.54 (9)
C25—Al—O1—C1	114.58 (15)	C3—C2—C9—C10	−60.76 (11)
C21—Al—O1—C1	−111.00 (15)	C1—C2—C9—C10	59.34 (11)
Al^i^—O2—C1—O1	24.1 (3)	C14—C9—C10—C11	1.83 (15)
Al^i^—O2—C1—C2	−158.73 (15)	C2—C9—C10—C11	177.43 (9)
Al—O1—C1—O2	−12.9 (2)	C9—C10—C11—C12	−0.82 (16)
Al—O1—C1—C2	169.90 (11)	C10—C11—C12—C13	−0.44 (17)
O2—C1—C2—C9	0.80 (13)	C11—C12—C13—C14	0.67 (18)
O1—C1—C2—C9	178.13 (8)	C10—C9—C14—C13	−1.60 (16)
O2—C1—C2—C15	−120.15 (10)	C2—C9—C14—C13	−177.00 (10)
O1—C1—C2—C15	57.18 (11)	C12—C13—C14—C9	0.37 (17)
O2—C1—C2—C3	118.74 (10)	C9—C2—C15—C20	121.13 (11)
O1—C1—C2—C3	−63.94 (11)	C3—C2—C15—C20	0.05 (13)
C9—C2—C3—C4	124.93 (10)	C1—C2—C15—C20	−119.09 (10)
C15—C2—C3—C4	−110.63 (11)	C9—C2—C15—C16	−62.24 (12)
C1—C2—C3—C4	4.38 (13)	C3—C2—C15—C16	176.68 (9)
C9—C2—C3—C8	−52.35 (11)	C1—C2—C15—C16	57.54 (11)
C15—C2—C3—C8	72.08 (11)	C20—C15—C16—C17	−0.52 (16)
C1—C2—C3—C8	−172.91 (9)	C2—C15—C16—C17	−177.30 (10)
C8—C3—C4—C5	−0.80 (16)	C15—C16—C17—C18	0.15 (17)
C2—C3—C4—C5	−178.08 (10)	C16—C17—C18—C19	0.25 (18)
C3—C4—C5—C6	0.48 (18)	C17—C18—C19—C20	−0.28 (19)
C4—C5—C6—C7	−0.07 (18)	C16—C15—C20—C19	0.49 (16)
C5—C6—C7—C8	0.01 (18)	C2—C15—C20—C19	177.11 (10)
C6—C7—C8—C3	−0.36 (18)	C18—C19—C20—C15	−0.10 (18)
C4—C3—C8—C7	0.74 (16)	Al—C21—C22—C24	−66.22 (12)
C2—C3—C8—C7	178.19 (10)	Al—C21—C22—C23	170.40 (8)
C15—C2—C9—C14	−10.07 (13)	Al—C25—C26—C28	58.20 (13)
C3—C2—C9—C14	114.64 (10)	Al—C25—C26—C27	−178.14 (9)
C1—C2—C9—C14	−125.27 (10)		

Symmetry code: (i) −*x*+1, −*y*+1, −*z*+1.
