# Supplementary material for: Crystal structure of bis(μ2-tri­phenyl­acetato-κO:κO′)bis(diisobutylaluminium)
Source: Acta Crystallogr E Crystallogr Commun. 2019 Mar 15;75(Pt 4):456–9. doi: 10.1107/S2056989019003396 (PMC6509680; doi:10.1107/S2056989019003396)
Supplement: Supplementary file 2 [file e-75-00456-sup4.pdf]

Acta Cryst E

STRUCTURE
REPORTS

Volume 75 (2019)

Supporting information for article:

Crystal structure of bis(μ2-triphenylacetato-κ*O*:κ*O′*)bis(diisobutylaluminium)

Alexander A. Vinogradov, Mikhail E. Minyaev, Konstantin A. Lyssenko and Ilya E. Nifant'ev

Parameters for two non-valence intermolecular interactions of H_{Ph} atoms with the π-system of a Ph-group (H6..C12^{ii} and H7..C11^{ii}) are given in Table S1 [symmetry code: (ii) −*x*+2, −*y*+2, −*z*+2]. Interacting Ph-rings are located nearly perpendicular with the corresponding angle between C3..C8 and C9^{ii}..C14^{ii} planes being of 82.83(3)°. The packing plots below demonstrate the impact of these C-H_{Ph}..C_{Ph} interactions among the 2/3^{rds} of phenyl groups on crystal packing, leading to orthogonal orientations of these Ph groups.

Table S1. Hydrogen-bond geometry (Å, °)

D—H···*A*	*D*—H	H···*A*	*D*···*A*	*D*—H···*A*
C6—H6···C12^{ii}	0.95	2.89	3.6598 (16)	138.7
C7—H7···C11^{ii}	0.95	2.98	3.7509 (16)	139.3

Symmetry code: (ii) −*x*+2, −*y*+2, −*z*+2.

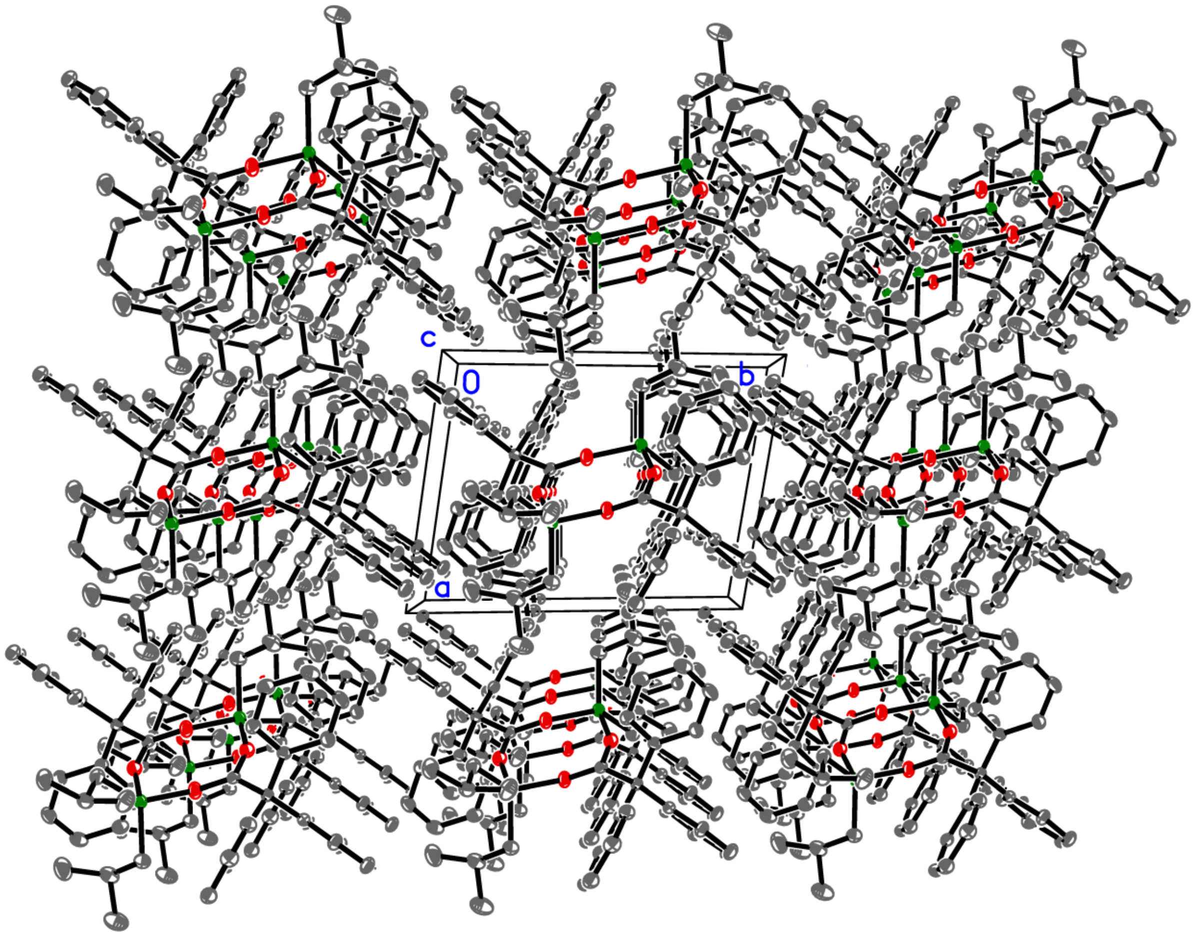

Figure S1. A packing plot of $[Al(^{i}Bu)_2(O_2CCPh_3)]_2$ parallel to the *ab* plane H-atoms are omitted for clarity. Displacement ellipsoids are set to the 50% probability level.

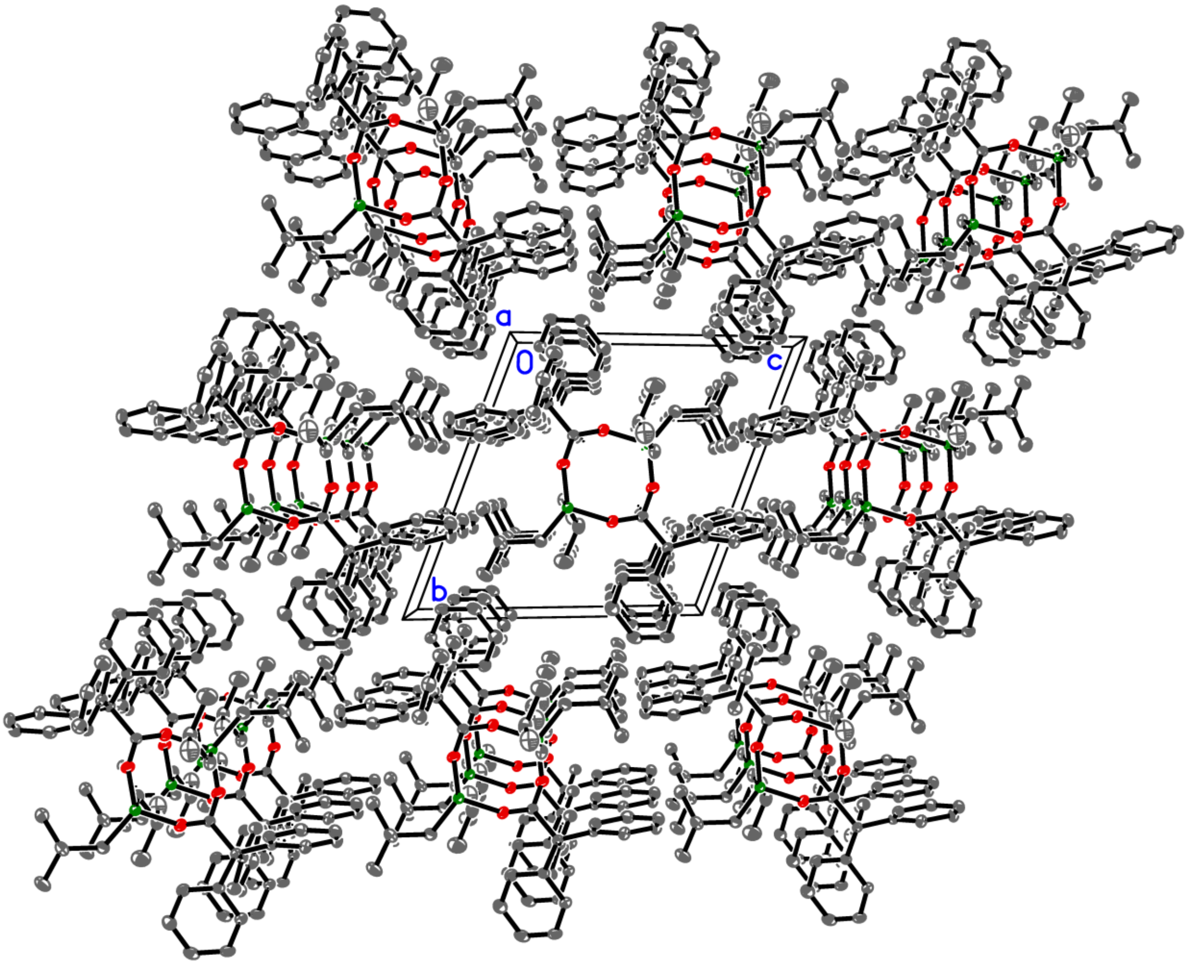

Figure S2. A packing plot of $[Al(^{i}Bu)_2(O_2CCPh_3)]_2$ parallel to the *ab* plane. H-atoms are omitted for clarity. Displacement ellipsoids are set to the 50% probability level.

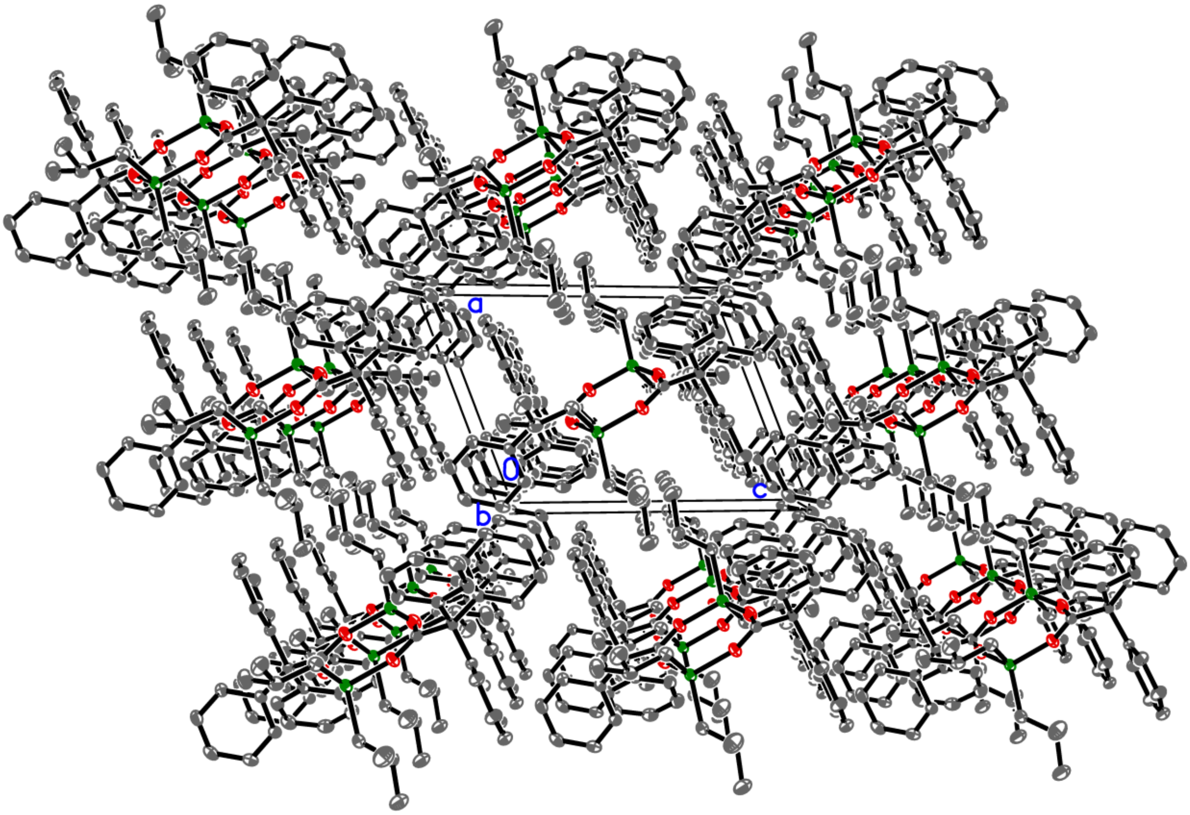

Figure S3. A packing plot of [Al(^{i}Bu)$_2$(O_2CCPh_3)]$_2$ parallel to the *ac* plane. H-atoms are omitted for clarity. Displacement ellipsoids are set to the 50% probability level.
